# Rotavirus NSP4 is secreted from infected cells as an oligomeric lipoprotein and binds to glycosaminoglycans on the surface of non-infected cells

**DOI:** 10.1186/1743-422X-8-551

**Published:** 2011-12-20

**Authors:** Alicia Didsbury, Carol Wang, Daniel Verdon, Mary A Sewell, Julie D McIntosh, John A Taylor

**Affiliations:** 1 School of Biological Sciences, University of Auckland, Auckland, New Zealand; 2 Maurice Wilkins Centre for Molecular Biodiscovery, University of Auckland, Auckland, New Zealand

## Abstract

**Background:**

Nonstructural glycoprotein 4 (NSP4) encoded by rotavirus is the only viral protein currently believed to function as an enterotoxin. NSP4 is synthesized as an intracellular transmembrane glycoprotein and as such is essential for virus assembly. Infection of polarized Caco-2 cells with rotavirus also results in the secretion of glycosylated NSP4 apparently in a soluble form despite retention of its transmembrane domain. We have examined the structure, solubility and cell-binding properties of this secreted form of NSP4 to further understand the biochemical basis for its enterotoxic function. We show here that NSP4 is secreted as discrete detergent-sensitive oligomers in a complex with phospholipids and demonstrate that this secreted form of NSP4 can bind to glycosaminoglycans present on the surface of a range of different cell types.

**Methods:**

NSP4 was purified from the medium of infected cells after ultracentrifugation and ultrafiltration by successive lectin-affinity and ion exchange chromatography. Oligomerisation of NSP4 was examined by density gradient centrifugation and chemical crosslinking and the lipid content was assessed by analytical thin layer chromatography and flame ionization detection. Binding of NSP4 to various cell lines was measured using a flow cytometric-based assay.

**Results:**

Secreted NSP4 formed oligomers that contained phospholipid but dissociated to a dimeric species in the presence of non-ionic detergent. The purified glycoprotein binds to the surface of various non-infected cells of distinct lineage. Binding of NSP4 to HT-29, a cell line of intestinal origin, is saturable and independent of divalent cations. Complementary biochemical approaches reveal that NSP4 binds to sulfated glycosaminoglycans on the plasma membrane.

**Conclusion:**

Our study is the first to analyze an authentic (i.e. non-recombinant) form of NSP4 that is secreted from virus-infected cells. Despite retention of the transmembrane domain, secreted NSP4 remains soluble in an aqueous environment as an oligomeric lipoprotein that can bind to various cell types via an interaction with glycosaminoglycans. This broad cellular tropism exhibited by NSP4 may have implications for the pathophysiology of rotavirus disease.

## Background

Rotaviruses infection causes acute watery diarrhea predominantly in infants of a wide range of animal species including humans. The virus is transmitted *via *the fecal-oral route and replication occurs predominantly within terminally differentiated epithelial cells located at the villous tips of the small intestine [1]. Symptoms of rotavirus infection are underpinned by several distinct pathophysiological mechanisms; malabsorption due to virus destruction of mature enterocytes, a decrease in epithelial permeability and a secretory component mediated by a virus-encoded enterotoxin. The enterotoxic activity has been attributed to NSP4, a non-structural glycoprotein released from rotavirus-infected cells [2,3]. Rotavirus appears to be unique among enteric viruses in the production of an enterotoxin, whose pathophysiological role may be analogous to the many well-characterized toxins produced by enteric bacterial pathogens like *Vibrio cholera*.

In addition to an (extracellular) enterotoxic function, siRNA knockdown of NSP4 demonstrates an essential role in virion morphogenesis within rotavirus-infected cells where the protein is localised to discrete membraneous domains that surround viroplasmic inclusions [4,5]. NSP4 is critically involved in the budding of newly formed double-layered particles as they enter the lumen of ER-derived vesicles [5]. The topology of NSP4 is typical of a type II transmembrane glycoprotein with the majority of the polypeptide oriented in the cytoplasm, a single hydrophobic transmembrane anchor sequence and a short luminal domain containing two N-linked glycans [6]. Surprisingly, NSP4 can also be secreted from rotavirus infected Caco-2 cells without the proteolytic removal of the hydrophobic transmembrane region yet remains soluble in aqueous media [7,8] The active secretion of NSP4 from infected cells is consistent with its proposed enterotoxic function. However, the dual role of NSP4 as an intracellular transmembrane glycoprotein involved in virus assembly and as a secreted, soluble enterotoxin is paradoxical. To further understand the biochemical basis for distinct intra- and extracellular NSP4 functions we have purified the secreted form of the protein from the media of infected Caco-2 cells and here determine some key biochemical features. We show that NSP4 is secreted as discrete detergent-sensitive oligomers in a complex with phospholipids and demonstrate that this secreted form of NSP4 can bind to glycosaminoglycans present on the surface of a range of different cell types.

## Methods and materials

### Cells and viruses

The rhesus monkey kidney cell line MA104 and Caco-2 cells derived from human colonic epithelium were grown in Dulbecco's modified Eagle's medium (DMEM) (Invitrogen, Carlsbad, CA) supplemented with 10% fetal bovine serum (FBS). Bovine rotavirus (UK strain) was obtained from the late Ian Holmes, University of Melbourne and propagated in MA104 cells as described previously [7].

### Purification of NSP4

NSP4 was purified from the medium of Caco-2 cells infected with bovine rotavirus (UK strain) at a MOI = 10. At 36 hpi, the medium was removed, centrifuged at 100,000 × g for 2 h and concentrated ~15 fold in an Amicon flow cell (M.W. cut off = 10 kDa). NSP4 was purified from the concentrated medium by successive Concanavalin A affinity and cation exchange chromatography (Mono S) in the absence of detergent and was determined to be > 95% pure by SDS PAGE and silver staining.

### Lipid analysis

Lipid was extracted from purified NSP4 in a chloroform:methanol extraction as described in [9] and with ketone as the internal standard The final lipid extract was dried under nitrogen, the lipid resuspended in a minimal volume of chloroform and the NSP4-derived lipid and lipid standards each spotted on separate silica-coated S-III Chromarods. The Chromarods were developed for 28 min in 60 ml of Hexane, 6 ml of Diethyl-ether and 0.1 ml of Formic Acid, dried for 5 min in a Rod Dryer TK-8 (Iatron Laboratories) at 60°C, then run in the Iatroscan Mark V^new ^Thin Layer Chromatography/Flame Ionization Detection (TLC/FID) system (Iatron, Japan) with a 30 s scan and settings of 2,000 ml min-1 O_2 _and 160 ml min-1 H_2_. Data were collected with an SESChromstar PC-board and the peaks quantified using SES-Chromstar version 4.10 (SES Analysesysteme).

### Measurement of NSP4 binding to cells

Cell suspensions were incubated with various concentrations of NSP4 for 30 min at 4°C. Cells were then pelleted by centrifugation and washed extensively in phosphate buffered saline (PBS) before being incubated with a purified monoclonal antibody specific for NSP4 (B4-2/55) [10]. After further washes, cells were incubated with fluorescent anti-mouse IgG and bound NSP4 was detected by flow cytometry using a FACSCalibur™ platform (BD Sciences) and CellQuest™ software. Viable cells were gated by their cell-specific forward and side scatter and at least 10,000 gated events were measured for each sample. NSP4 binding was quantified by measurement of the change in geometric mean fluorescent intensity (MFI) of cells incubated with and without NSP4. Statistical significance for all assays was determined using a two-tailed unpaired Student's *t*-test where *p *< 0.05 was considered statistically significant.

## Results

### Purification of secreted NSP4 by lectin-affinity and ion exchange chromatography

**Figure 1:**
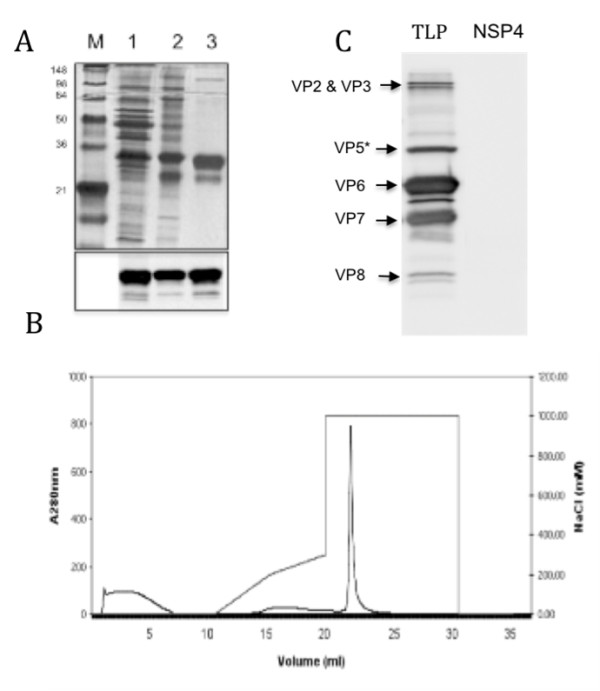
**Purification of NSP4 from culture medium of rotavirus infected Caco-2 cells**. NSP4 was purified by concanavalin A affinity chromatography and cation exchange (Mono S) chromatography. A, Purification was monitored by SDS PAGE and silver staining of electrophoresed proteins Lane 1, M.W. markers; 2, concentrated media post centrifugation; 3, protein eluted from conA in 1 M α-methyl mannoside; 4, Mono S eluate (1 M NaCl). B, Elution profile of material loaded on Mono S column is shown in B. C, Western blot of purified rotavirus TLPs (2.5 μg) and purified NSP4 3 μg probed with a polyclonal antiserum (D37) raised against purified SA11 rotavirus

NSP4 is secreted from rotavirus-infected Caco-2 cells as a full-length (i.e. non- proteolytically processed) protein [7]. To investigate the biophysical properties of the secreted protein we first sought to purify NSP4 from the medium of Caco-2 cells infected with bovine (UK strain) rotavirus. Medium was recovered from cells at 36 hpi at which time cell viability was ~75% but the yield of secreted protein was significantly greater than at 24 hpi when the viability was greater than 90% [7]. The medium was ultracentrifuged to remove virions, concentrated and NSP4 purified by successive lectin-affinity and ion-exchange chromatography (Figure 1). Purity was confirmed by SDS PAGE and silver staining and the absence of contaminating virus proteins was confirmed by immunoblotting with anti-rotavirus antibody (Figure 1c). The presence of a smaller band at ~ 26 kDa is likely due to proteolytic degradation given previous reports that the C-terminal domain of NSP4 is susceptible to proteolysis [11].

### Secreted NSP4 forms detergent-sensitive oligomers

**Figure 2:**
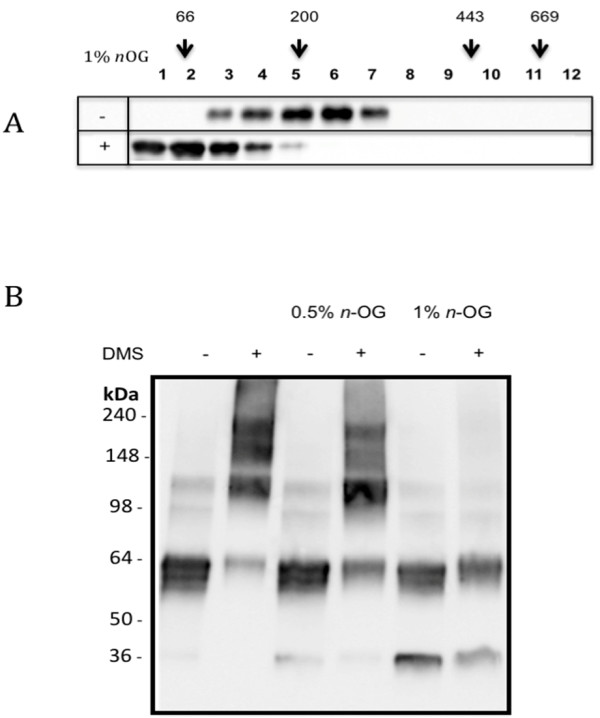
**Analysis of purified NSP4 by velocity gradient centrifugation and cross-linking with the bi-functional reagent dimethyl suberimidate (DMS)**. (A) Purified NSP4 was loaded on a 5-20% sucrose gradient and centrifuged for 15 h at 100,000 × g. The gradient was fractionated from the top and the presence of NSP4 determined by western blotting. Identical gradients centrifuged in parallel were used to determine the migration BSA (66 kDa), β-amylase (200 kDa), ferritin (443 kDa) and thyroglobulin (669 kDa). A second series of gradients were run in the presence of 1% *n*-octyl glucoside (lower panel - A). (B) NSP4 was incubated with 5 mM dimethyl suberimidate (DMS) for 60 min and the stabilisation of oligomeric species analysed by SDS PAGE and western blotting. Protein was crosslinked for 60 min at room temperature, samples were run on SDS PAGE without boiling and NSP4 detected by western blotting

We previously detected the presence of N-linked glycans located at residues 8 and 18 suggests in the secreted NSP4 indicating that the protein is not processed at the N-terminus and therefore retains the hydrophobic transmembrane domain located between residues 27 and 44 [7]. We hypothesized that the secreted NSP4 might form oligomers to maintain the solubility of the protein in an aqueous environment. NSP4 was analyzed by velocity gradient centrifugation in parallel with a series of proteins of known molecular weight that were applied to identical gradients. The gradients were fractioned identically and the distribution of NSP4 analyzed by western blot and compared to the migration of protein standards (Figure 2a). The majority of NSP4 was recovered from the middle of the gradient corresponding to a molecular weight of ~240 kDa. When the experiment was repeated in the presence of the non-ionic detergent *n*-octyl glucoside (1% w/v) the migration of NSP4 in the gradient was reduced, consistent with a reduction in the native molecular weight to ~60 kDa. Given that the monomeric molecular weight is 28 kDa, the data suggest that the secreted form of the protein forms an oligomer. The oligomeric structure of NSP4 was further analyzed using the bifunctional reagent dimethyl suberimidate (DMS) to stabilize oligomeric species prior to SDS PAGE. 5 mM DMS stabilized discrete oligomeric forms of NSP4 that were apparent following SDS PAGE (Figure 2b). This approach revealed the presence of tetrameric, hexameric and octameric species of NSP4 stabilized by the crosslinker and an SDS-resistant dimeric form. Incubation of the protein for 30 min in 1% *n*-octyl glucoside prior to addition of DMS inhibited the formation of any oligomeric species greater than dimers but when the concentration of detergent was reduced to 0.5%, i.e. below the critical micellar concentration, there was no effect on oligomerisation. Taken together, these results strongly suggest that the NSP4 secreted from infected Caco-2 cells is an oligomer, formed by the assembly of detergent-resistant dimers.

**Figure 3:**
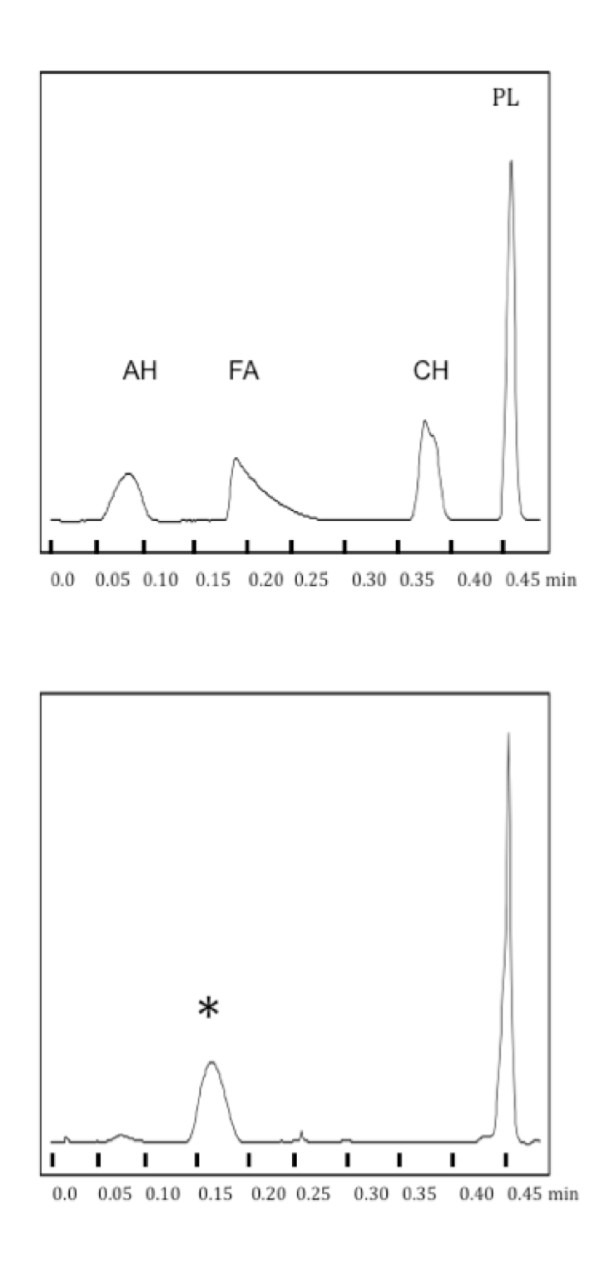
**NSP4 is secreted as complex with phospholipid**. TLC/FID chromatograms of lipids extracted from purified NSP4, compared to standards (upper panel; AH = aliphatic hydrocarbon, FA = free fatty acid, Ch = cholesterol, PL = phospholipid). The major peak in the NSP4-derived sample corresponds to phospholipid. The minor peak marked *represents the ketone internal standard added to the NSP4 sample to permit quantification of the extracted lipid

The effect of detergent on the structure of NSP4 could also indicate a role for lipids in maintaining the solubility and oligomeric structure of the secreted glycoprotein. To determine whether NSP4 was secreted in a complex with lipid the purified protein was extracted with chloroform/methanol and the organic phase analyzed by thin-layer chromatography and flame ionization detection (TLC-FID) using an Iatroscan [9]. This approach revealed a significant phospholipid peak in the extracted organic phase (Figure 3), indicating that NSP4 is secreted from rotavirus-infected cells as a lipoprotein.

### Saturable binding of NSP4 to HT29 cells is independent of divalent cations

**Figure 4:**
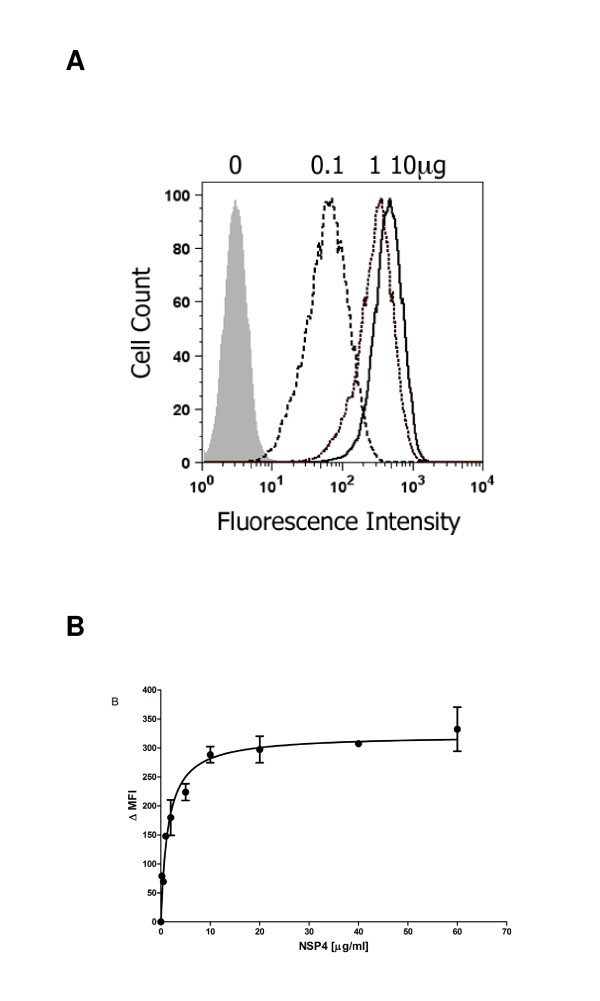
**Saturable binding of NSP4 to HT29 cells**. A, Histogram profiles of NSP4 (0.1, 1.0 and 10 μg/ml) binding to HT-29 cells. One representative of three independent experiments is shown. B, Saturable binding of NSP4: changes in mean fluorescent intensity relative to cells stained with primary and secondary antibody in the absence of NSP4 (ΔMFI) versus NSP4 concentration. Data are mean and SEM of duplicate samples, from three independent experiments

**Figure 5:**
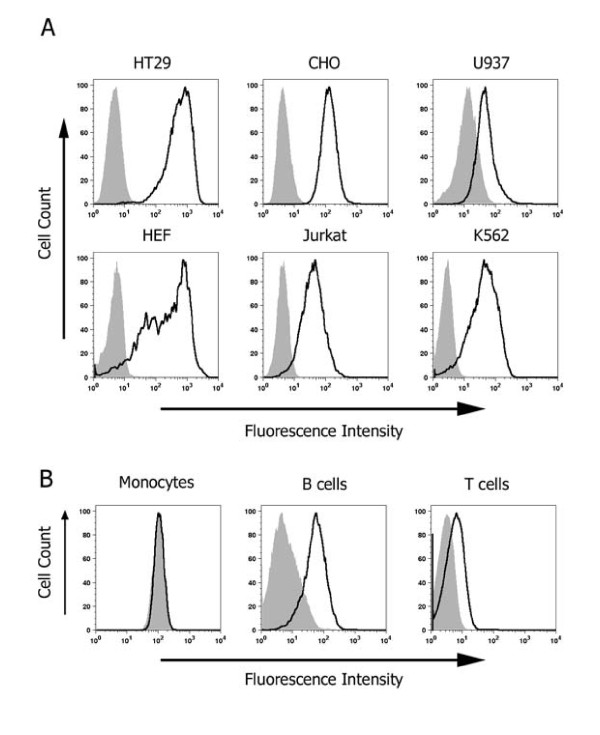
**NSP4 binds to cells of distinct lineage**. A, Cells were incubated with 5 μg/ml NSP4 and binding measured as described in the text. A representative histogram from three independent experiments is shown. Shaded peak indicates background fluorescence of cells stained with primary and secondary antibody in the absence of NSP4. B, Human peripheral blood mononuclear cells (PBMC) were incubated with 5 μg/ml NSP4 and binding measured as described in the text. The PBMC were stained with antibodies directed against CD3, CD20 and CD14 to identify T cell, B cell and monocyte subsets respectively

**Figure 6:**
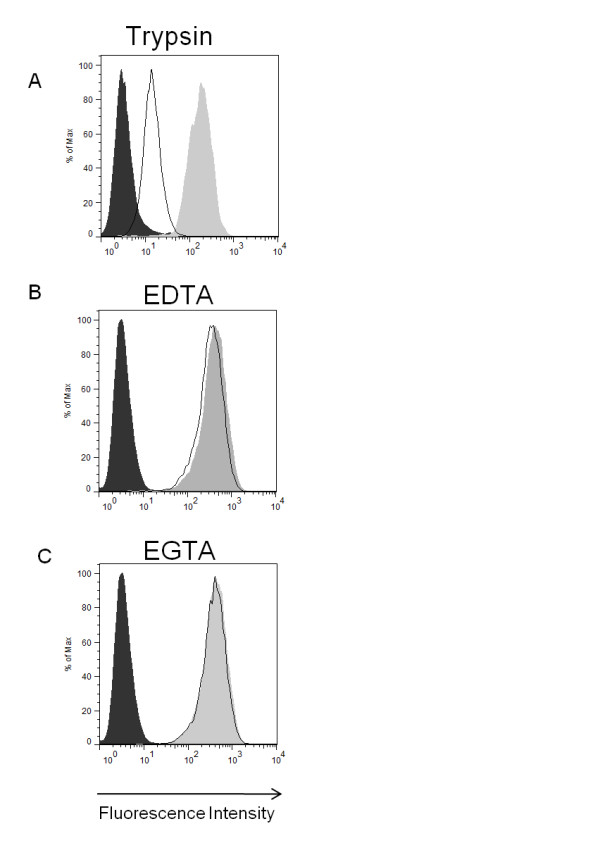
**NSP4 binds to HT-29 cells via a trypsin-sensitive receptor but binding does not require divalent cations**. Trypsin-treated HT-29 cells (A) were incubated with 5 μg/ml NSP4 and binding analyzed as described in text. The requirement for divalent cations was examined by inclusion of 10 mM EDTA (B) or EGTA (C) in the binding assay. In each case the dark shaded peak indicates background fluorescence of cells stained with primary and secondary antibody in the absence of NSP4, light shaded peak, cells incubated with NSP4 and the unshaded peak cells incubated with NSP4 plus treatment

The extracellular form of NSP4 can function as an enterotoxin during rotavirus infection causing diarrhea in neonatal mice [12]. In common with other microbial enterotoxins, NSP4 should therefore be recruited to the surface of cells and interact with a receptor prior to triggering a signaling cascade that contributes to diarrhea. Therefore we examined whether the secreted NSP4 was capable of binding to the surface of human intestinal epithelial HT-29 cells using a flow cytometry-based assay. Binding was saturated at concentrations above 10 μg/ml suggesting the existence of a receptor and exhibited a Kd of 1.4 μg/ml (Figure 4b). NSP4 bound also to the surface of several other epithelial and hematopoetic transformed cell lines, to human embryonic fibroblasts (HEF) and to human B cells but bound only weakly or not at all to primary T cells and monocytes (Figure 5). Significant variation in the amount of NSP4 bound by different cell types was observed; for example the amount bound by HT29 was approx 500 times greater than by U937 cells. Binding of NSP4 to HT29 cells was reduced significantly when cells were pre-treated with trypsin (0.025% w/v for 15 min at 37°C) and then washed extensively (Figure 6). By contrast the inclusion of EDTA or EGTA in the binding assay did not significantly reduce NSP4 binding. This result is in contrast to the results of a previous study that demonstrated the requirement for Mg^2+ ^or Mn^2+ ^ions in the binding of NSP4 to an immobilized integrin I domain [13].

#### NSP4 binds to glycosaminoglycans

**Figure 7:**
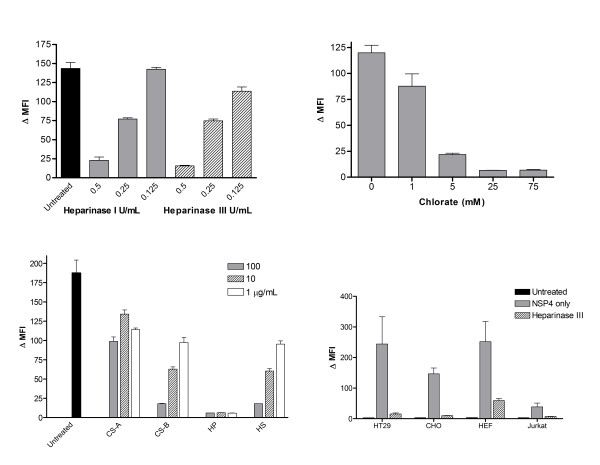
**NSP4 binds to cells via glycosaminoglycan receptors**. A, Binding of NSP4 to HT-29 cells treated with Heparinase I or III. B, Pre-treatment of CHO cells with sodium chlorate inhibits the binding of NSP4. Cells were incubated with various concentrations of chlorate for 24 h prior to suspension and addition to the binding assay. C. Inhibition of NSP4 binding by inclusion of soluble GAGs in the binding assay; CS-A; chondroitin sulphate A, CS-B; chondroitin sulphate B, HP; heparin, HS; heparan sulphate. D, Inhibition of NSP4 binding by Heparinase III treatment in different cell lines. Data shown are mean +/- SEM from at least three independent experiments

The ability of NSP4 to bind to cells of diverse origin and the effect of trypsin treatment suggests that the receptor(s) involved might be a ubiquitously expressed protein or protein-containing plasma membrane component. Glycosaminoglycans (GAGs) are present on the plasma membrane of most mammalian cells and are frequently used as receptors by viruses and viral proteins. The importance of GAGs in NSP4 binding was first investigated using specific heparin lyases. Heparinase I cleaves heparin and, with lower specificity, heparan sulfate, while the primary substrate for Heparinase III is heparan sulfate. Suspensions of HT-29 cells were treated with varying amounts of Heparinase I or III, for 60 min at 37°C. The digested cells were washed three times in cold DMEM, before incubation with 5 μg/ml NSP4 on ice and binding analyzed by flow cytometry (Figure 7a). Treatment with either Heparinase I or III at concentrations greater than 0.25 U/ml reduced binding by 50-90% compared to a control, (*p *< 0.05). At an enzyme concentration of 0.125 U/ml, Heparinase I treatment did not significantly reduce binding (*p *= 0.9) whereas Heparinase III decreased binding by 20% (*p *< 0.05), suggesting that heparan sulfate might be the principal GAG involved in binding NSP4.

To confirm the role of GAGs in the binding of NSP4 and whether GAGs mediate binding of NSP4 to multiple cell types, binding assays were carried out in the presence of soluble heparin, heparan sulfate, chondroitin sulfate A (CSA) or, chondroitin sulfate B, (CSB). CSA was the least effective inhibitor of the four GAGs, with a 45% reduction in binding observed at 100 μg/ml (*p *< 0.05). CSB inhibited NSP4 binding at all concentrations, with 50% reduction (*p *< 0.01) observed in concentrations as low as 1 μg/ml (Figure 7b). Both heparan sulfate and heparin reduced binding of NSP4 in a dose-dependent manner. 100 μg/ml heparan sulfate reduced binding by almost 90% (*p *< 0.001), while the presence of 10 μg/ml heparin reduced the level of binding that of HT29 control cells without NSP4 (*p *< 0.001). Soluble heparin was able to inhibit binding of NSP4 to a range of different cell types indicating that GAGs could potentially recruit NSP4 to cells of distinct lineage (Figure 7c).

### GAG sulfation is critical for NSP4 binding

The biochemical diversity of GAGs is increased by cell-specific modifying enzymes. In particular sulfotransferase enzymes influence the position and degree of sulfation, a major determinant of specificity in protein-GAG interactions [14]. To investigate the possible role of sulfation in NSP4 binding, cells were pretreated with sodium chlorate, an inhibitor of sulfate adenylyltransferase, for 24 h resulting in decreased sulfation of cellular proteins and carbohydrates [14]. CHO cells were used for this experiment due to the toxicity of chlorate for HT-29. Cells were incubated in increasing concentrations of sodium chlorate prior to incubation with 5 μg/ml NSP4 for 1 h on ice, and bound protein measured. Treatment with sodium chlorate abolished NSP4 binding in a dose-dependant manner, indicating an absolute requirement for GAG sulfation (Figure 7d).

## Discussion

A direct role for NSP4 in the acute watery diarrhea caused by rotavirus infection was first proposed over a decade ago following the observation that a peptide derived from the cytoplasmic domain of the protein caused diarrhea in 3 day old mice when injected intra-illeally or intra peritoneally [12]. These studies led to the identification of NSP4 as a functional enterotoxin, the first, and currently only such virus-encoded protein known to exhibit this property. Subsequent *in vitro *experiments confirmed that NSP4 could potentiate secretion of Cl^- ^ions and water from isolated mouse crypts through PLC-dependant elevation of cytosolic Ca^2+ ^[3,15]. Notably, these physiological studies utilized synthetic peptides or recombinant forms of NSP4 purified from detergent-solubilized insect cells and, preceded direct evidence that NSP4 was secreted from rotavirus infected cells, a prerequisite likely to enable the protein to interact with the plasma membrane of intestinal epithelial or other physiologically relevant cells.

Rotavirus infection of non-differentiated cells is cytolytic with cell viability rapidly decreasing after as little as 8 h post infection. In the monkey kidney line MA104, a peptide corresponding to residues 112-175 of NSP4 was released into the medium prior to cell lysis and recombinant forms of this peptide were capable of causing diarrhea in mice when purified from insect cells [16]. In contrast, infection of differentiated Caco-2 cells, a polarized cell line derived from colonic epithelia, support non-lytic infection in which rotavirus is actively secreted from the apical surface with cell viability only slightly decreased after 72 h [17]. We reported apical secretion of NSP4 from rotavirus infected Caco-2 cells as an intact species without proteolytic cleavage [7]. Secretion of the unprocessed fully glycosylated form of NSP4 was surprising given the presence of a transmembrane domain but has been confirmed recently by Gibbons et al., [8]. The results presented here indicate that NSP4 is secreted as an oligomeric lipoprotein in complex with phospholipid. The affinity of NSP4 for negatively charged phospholipids and cholesterol has been reported previously [18]. We propose that recruitment of lipids during oligomerisation of NSP4 subunits in the ER membrane or a post ER membrane membraneous compartment facilitates its extrusion from the bilayer and release from infected cells as a small lipoprotein particles.

Purification of NSP4 to homogeneity from the media of rotavirus-infected cells confirmed our previous demonstration that the glycoprotein is not released in membrane vesicles like exosomes that contain additional proteins. Biophysical studies reveal that the secreted NSP4 is homogeneous in size. Previous studies have demonstrated a tetrameric structure for soluble forms of truncated regions of the cytoplasmic domain of NSP4 following recombinant expression or chemical synthesis [19-21]. The experiments reported here are, to our knowledge, the first that address the biophysical form of the full-length protein produced in rotavirus-infected cells. Although we have tentatively assigned a hexameric or octameric structure to the oligomer on the basis of apparent MW and crosslinking data, these data may also reflect an elongated polypeptide conformation and/or the ability to form higher order oligomers through weak hydrophobic interactions and thus we do not exclude that full length NSP4 is tetrameric.

To function as an enterotoxin, NSP4 should bind to a range of target cells and activate signaling pathways *via *receptors in the plasma membrane. A study in rotavirus-infected mice revealed that NSP4 was located predominantly on the basement membrane of villous epithelia that were not directly infected and identified fibronectin as a putative receptor [22]. More recently, Seo et al., demonstrated that the metal ion-dependent adhesion site (MIDAS) motif present on integrins α1β1 and α2β1 can function as a receptor for NSP4 on cultured cells [13]. All previous studies have employed NSP4 produced in either recombinant bacteria or insect cell lines [23-25]. Therefore, the present study was carried out to establish whether the native, full-length form of NSP4 secreted from polarized mammalian cells infected with rotavirus, is able to bind to non-infected mammalian cells and whether putative receptors can be identified.

The range of cells to which NSP4 binds includes cells of epithelial, fibroblast and hematopoetic origin, though considerable variation in the amount of NSP4 binding was observed between different cell types. The degree of GAG sulfation is a critical determinant of NSP4 binding revealed by the effect of chlorate treatment and the fact that HP, HS and CSB, the most highly sulfated GAGs used in our experiments, exhibited the greatest ability to inhibit binding of NSP4 to cells. These results could indicate that NSP4 (pI = 8.4), binds primarily *via *electrostatic interactions and that spacing of sulfate groups rather distinct sugar residues is a critical determinant for binding. Our experiments do not define a precise GAG structure targeted by NSP4 but the effect of heparanase treatment strongly suggests that heparan sulphate is the major GAG species required for binding to HT-29 cells. The specificity of NSP4-GAG interactions could be further explored using engineered cell lines that lack specific proteoglycan forms or overexpress different sulfotransferase enzymes that are required to generate highly sulfated GAG structures.

Many viral and host molecules interact with GAGs on the surface of cells. Viruses including herpes simplex-1 (HSV-1), human papillomavirus, dengue virus, and human immunodeficiency virus engage HS on the surface of cells during the initial stages of infection [26-29]. For example, HSV-1 initiates infection by attaching to HS on the cell surface via its surface glycoprotein gB and/or gC. A third viral glycoprotein gD can then interact with a specific form of HS known as 3-O-sulfated heparan sulphate to trigger membrane fusion and viral entry [28]. Our results are also consistent with the behavior of a nonstructural glycoprotein encoded by dengue viruses. NS1 secreted from cells infected with dengue virus can utilize HS and CSE to attach to the surface of various cells types and this interaction may be a factor in the vascular leakage associated with secondary dengue virus infection [30]. It is unlikely that the ability of NSP4 to activate intracellular signaling in intestinal and potentially other cells types is directly mediated by an interaction with GAGs. Rather GAGs may serve to recruit and tether NSP4 to the surface of cells enabling its interact with additional specific receptors to activate signaling pathways [31]. While the primary focus of NSP4 has been on its role as an enterotoxin and its pathophysiological effects on intestinal cells, our studies now reveal that the protein may have a broader cellular tropism and thus exert a wider range of molecular effects in the host of relevance to rotavirus disease.

## Conclusion

Our study is the first to purify and analyse biochemical properties of an authentic (i.e. non-recombinant) form of NSP4 that is secreted from virus-infected cells. Although retaining the hydrophobic transmembrane domain, the secreted glycoprotein remains soluble in an aqueous environment as an oligomeric lipoprotein. NSP4 bound to various cell types via interaction with sulfated glycosaminoglycan receptors. The broad cellular tropism exhibited by NSP4 may have implications for the pathophysiology of rotavirus disease.

## Abbreviations

GAG: Glycosaminoglycan; HS: Heparin sulfate; CS-A: Chondroitin sulfate A; CS-B: Chondroitin sulfate B; PLC: Phospholipase C; MFI: Mean fluorescent intensity; hpi: Hours post infection.

## Competing interests

The authors declare that they have no competing interests.

## Authors' contributions

JAT designed the research; AD, CW, JM, DV performed the research; AD, CW, JM, DV, MS and JAT analyzed the data; JAT wrote the manuscript. All authors read and approved the final manuscript.

## References

[B1] Greenberg HB Estes MK Rotaviruses: from pathogenesis to vaccination Gastroenterology 2009 136 1939 1951 10.1053/j.gastro.2009.02.076 19457420 PMC3690811

[B2] Ball JM Mitchell DM Gibbons TF Parr RD Rotavirus NSP4: a multifunctional viral enterotoxin Viral Immunol 2005 18 27 40 10.1089/vim.2005.18.27 15802952

[B3] Morris AP Scott JK Ball JM Zeng CQ O'Neal WK Estes MK NSP4 elicits age-dependent diarrhea and Ca(2+)mediated I(-) influx into intestinal crypts of CF mice Am J Physiol 1999 277 G431 G444 10444458 10.1152/ajpgi.1999.277.2.G431

[B4] Silvestri LS Tortorici MA Vasquez-Del Carpio R Patton JT Rotavirus glycoprotein NSP4 is a modulator of viral transcription in the infected cell J Virol 2005 79 15165 15174 10.1128/JVI.79.24.15165-15174.2005 16306588 PMC1316041

[B5] Lopez T Camacho M Zayas M Najera R Sanchez R Arias CF Lopez S Silencing the morphogenesis of rotavirus J Virol 2005 79 184 192 10.1128/JVI.79.1.184-192.2005 15596814 PMC538724

[B6] Bergmann CC Maass D Poruchynsky MS Atkinson PH Bellamy AR Topology of the non-structural rotavirus receptor glycoprotein NS28 in the rough endoplasmic reticulum EMBO J 1989 8 1695 1703 2548854 10.1002/j.1460-2075.1989.tb03561.x PMC401011

[B7] Bugarcic A Taylor JA Rotavirus nonstructural glycoprotein NSP4 is secreted from the apical surfaces of polarized epithelial cells J Virol 2006 80 12343 12349 10.1128/JVI.01378-06 17035333 PMC1676281

[B8] Gibbons TF Storey SM Williams CV McIntosh A Mitchel DM Parr RD Schroeder ME Schroeder F Ball JM Rotavirus NSP4: Cell type-dependant transport kinetics to the exofacial plasma membrane and release from intact cells Virol J 2011 8 278 10.1186/1743-422X-8-278 21645398 PMC3129587

[B9] Sewell MA Utilization of lipids during early development of the sea urchin Evechinus chloroticus Marine Ecology-Progress Series 2005 304 133 142

[B10] Hyser JM Zeng CQ Beharry Z Palzkill T Estes MK Epitope mapping and use of epitope-specific antisera to characterize the VP5* binding site in rotavirus SA11 NSP4 Virology 2008 373 211 228 10.1016/j.virol.2007.11.021 18164740 PMC2377065

[B11] O'Brien JA Taylor JA Bellamy AR Probing the structure of rotavirus NSP4: a short sequence at the extreme C terminus mediates binding to the inner capsid particle J Virol 2000 74 5388 5394 10.1128/JVI.74.11.5388-5394.2000 10799621 PMC110899

[B12] Ball JM Tian P Zeng CQ Morris AP Estes MK Age-dependent diarrhea induced by a rotaviral nonstructural glycoprotein Science 1996 272 101 104 10.1126/science.272.5258.101 8600515

[B13] Seo NS Zeng CQ Hyser JM Utama B Crawford SE Kim KJ Hook M Estes MK Inaugural article: integrins alpha1beta1 and alpha2beta1 are receptors for the rotavirus enterotoxin Proc Natl Acad Sci USA 2008 105 8811 8818 10.1073/pnas.0803934105 18587047 PMC3021436

[B14] Hoogewerf AJ Cisar LA Evans DC Bensadoun A Effect of chlorate on the sulfation of lipoprotein lipase and heparan sulfate proteoglycans. Sulfation of heparan sulfate proteoglycans affects lipoprotein lipase degradation J Biol Chem 1991 266 16564 16571 1885587

[B15] Dong Y Zeng CQ Ball JM Estes MK Morris AP The rotavirus enterotoxin NSP4 mobilizes intracellular calcium in human intestinal cells by stimulating phospholipase C-mediated inositol 1,4,5-trisphosphate production Proc Natl Acad Sci USA 1997 94 3960 3965 10.1073/pnas.94.8.3960 9108087 PMC20550

[B16] Zhang M Zeng CQ Morris AP Estes MK A functional NSP4 enterotoxin peptide secreted from rotavirus-infected cells J Virol 2000 74 11663 11670 10.1128/JVI.74.24.11663-11670.2000 11090165 PMC112448

[B17] Svensson L Finlay BB Bass D von Bonsdorff CH Greenberg HB Symmetric infection of rotavirus on polarized human intestinal epithelial (Caco-2) cells J Virol 1991 65 4190 4197 1649325 10.1128/jvi.65.8.4190-4197.1991 PMC248854

[B18] Huang H Schroeder F Estes MK McPherson T Ball JM Interaction(s) of rotavirus non-structural protein 4 (NSP4) C-terminal peptides with model membranes Biochemical Journal 2004 380 723 733 10.1042/BJ20031789 15012630 PMC1224213

[B19] Taylor JA O'Brien JA Yeager M The cytoplasmic tail of NSP4, the endoplasmic reticulum-localized non-structural glycoprotein of rotavirus, contains distinct virus binding and coiled coil domains EMBO J 1996 15 4469 4476 8887538 PMC452176

[B20] Bowman GD Nodelman IM Levy O Lin SL Tian P Zamb TJ Udem SA Venkataraghavan B Schutt CE Crystal structure of the oligomerization domain of NSP4 from rotavirus reveals a core metal-binding site J Mol Biol 2000 304 861 871 10.1006/jmbi.2000.4250 11124032

[B21] Jagannath MR Kesavulu MM Deepa R Sastri PN Kumar SS Suguna K Rao CD N- and C-terminal cooperation in rotavirus enterotoxin: novel mechanism of modulation of the properties of a multifunctional protein by a structurally and functionally overlapping conformational domain J Virol 2006 80 412 425 10.1128/JVI.80.1.412-425.2006 16352566 PMC1317517

[B22] Boshuizen JA Rossen JW Sitaram CK Kimenai FF Simons-Oosterhuis Y Laffeber C Buller HA Einerhand AW Rotavirus enterotoxin NSP4 binds to the extracellular matrix proteins laminin-beta3 and fibronectin J Virol 2004 78 10045 10053 10.1128/JVI.78.18.10045-10053.2004 15331737 PMC514988

[B23] Horie Y Nakagomi O Koshimura Y Nakagomi T Suzuki Y Oka T Sasaki S Matsuda Y Watanabe S Diarrhea induction by rotavirus NSP4 in the homologous mouse model system Virology 1999 262 398 407 10.1006/viro.1999.9912 10502518

[B24] Mori Y Borgan MA Ito N Sugiyama M Minamoto N Diarrhea-inducing activity of avian rotavirus NSP4 glycoproteins, which differ greatly from mammalian rotavirus NSP4 glycoproteins in deduced amino acid sequence in suckling mice J Virol 2002 76 5829 5834 10.1128/JVI.76.11.5829-5834.2002 11992013 PMC137017

[B25] Rodriguez-Diaz J Lopez-Andujar P Garcia-Diaz A Cuenca J Montava R Buesa J Expression and purification of polyhistidine-tagged rotavirus NSP4 proteins in insect cells Protein Expr Purif 2003 31 207 212 10.1016/S1046-5928(03)00166-9 14550638

[B26] Chen Y Maguire T Hileman RE Fromm JR Esko JD Linhardt RJ Marks RM Dengue virus infectivity depends on envelope protein binding to target cell heparan sulfate Nat Med 1997 3 866 871 10.1038/nm0897-866 9256277

[B27] Johnson KM Kines RC Roberts JN Lowy DR Schiller JT Day PM Role of heparan sulfate in attachment to and infection of the murine female genital tract by human papillomavirus J Virol 2009 83 2067 2074 10.1128/JVI.02190-08 19073722 PMC2643729

[B28] Shukla D Liu J Blaiklock P Shworak NW Bai X Esko JD Cohen GH Eisenberg RJ Rosenberg RD Spear PG A novel role for 3-O-sulfated heparan sulfate in herpes simplex virus 1 entry Cell 1999 99 13 22 10.1016/S0092-8674(00)80058-6 10520990

[B29] Tamura M Natori K Kobayashi M Miyamura T Takeda N Genogroup II noroviruses efficiently bind to heparan sulfate proteoglycan associated with the cellular membrane J Virol 2004 78 3817 3826 10.1128/JVI.78.8.3817-3826.2004 15047797 PMC374263

[B30] Avirutnan P Zhang L Punyadee N Manuyakorn A Puttikhunt C Kasinrerk W Malasit P Atkinson JP Diamond MS Secreted NS1 of dengue virus attaches to the surface of cells via interactions with heparan sulfate and chondroitin sulfate E PLoS Pathog 2007 3 e183 10.1371/journal.ppat.0030183 18052531 PMC2092380

[B31] Oh MJ Akhtar J Desai P Shukla D A role for heparan sulfate in viral surfing Biochem Biophys Res Commun 2010 391 176 181 10.1016/j.bbrc.2009.11.027 19909728 PMC2812628

